# Intellectual Performance and Educational Attainment of Mexican Adolescents in Poverty

**DOI:** 10.5964/ejop.v15i3.1542

**Published:** 2019-09-27

**Authors:** Joaquina Palomar-Lever, Amparo Victorio-Estrada

**Affiliations:** a Department of Psychology, Universidad Iberoamericana, Mexico City, Mexico; Maria Grzegorzewska University, Warsaw, Poland

**Keywords:** intellectual performance, adolescents, parenting, Mexico, poverty

## Abstract

The objective of this study was to determine factors that can predict the intellectual performance and educational attainment of adolescents living in poverty. Data of Mexican adolescents from rural and urban areas (*N* = 1,093, 55.8% male, 61% urban) and their mothers were analyzed. The data came from a probabilistic sample with national representativeness of beneficiary households of the governmental program to fight poverty *Oportunidades*. Mothers and children were surveyed separately at home using questionnaires. Results from structural equations modeling revealed that higher intellectual performance was determined by older age, higher maternal intellectual performance and education, more adequate parenting practices, fewer siblings, and less insecure neighborhoods. Higher educational attainment was predicted by older age, higher intellectual performance, and more psychological resources. Data explained 25% of the variance of intellectual performance, and 39% of the variance of educational attainment. Results are discussed regarding the possibility of enhancing intellectual performance and education.

Educational attainment has been proposed as the most important mechanism to promote social mobility (Faas, Benson, & Kaestle, 2013), and intellectual performance is probably the most influential factor to promote educational attainment (Firkowska-Mankiewicz, 2011; Subotnik, Olszewski-Kubilius, & Worrell, 2011). In this sense, children’s intellectual performance affects their life expectations and attainments and influences their future social position and success (Subotnik, Olszewski-Kubilius, & Arnold, 2003). However, the cycle of poverty and low educational attainment perpetuates itself because poverty adversely affects the children´s educational achievement (Donlan, Prescott, & Zaff, 2016) and hinders their progress toward higher education levels. It has been found that the more pervasive the poverty in their environment, and the earlier and the more prolonged the experience of poverty during their development, the more deleterious the effects of poverty on children´s educational outcomes (Chaudry & Wimer, 2016). Hence, the identification of predictors of the intellectual performance of youth who live in poverty is of social relevance in a country with high levels of social inequality like Mexico.

Intelligence has been defined as several interrelated intellectual abilities, organized as independent systems consisting of general abilities (*g* factor), and specific abilities, such as “crystallized” knowledge (Bolger, Mackey, Wang, & Grigorenko, 2014). Intelligence is no longer viewed as strictly genetically determined, but as determined by genetic as well as by environmental factors (Bolger et al., 2014; Subotnik et al., 2011; Tucker-Drop & Harden, 2012). That is, the interaction between genetic, brain, and evironmental factors creates the diversity of cognitive functions that are regarded as human intelligence: Genetic factors establish the basis for the neurocognitive systems and the interaction between genetic and environmental factors determines the individual variation in intelligence (Bolger et al., 2014).

Both the general and specific intellectual abilities determine the global intellectual achievement; however, the general intellectual abilities derive from genetic and environmental factors, whereas the specific abilities are consciously acquired (Subotnik et al., 2011). In this regard, genetic factors explain aproximately 50% of the individual variance of the general intelectual abilities; and the general intellectual abilities explain most of the individual differences in intellectual achievement, but they cannot explain the individual differences in specific cognitive domains, such as verbal, mathematical or musical ability (Tucker-Drop & Harden, 2012).

The cognitive abilities underlying the intellectual functions develop with age until they reach the complexity that characterizes adult intelligence (Qin et al., 2014). In this sense, early childhood is a critical period for neurocognitive development; but adolescence is also a crucial period for the development of intellectual abilities, because some brain structures, like the prefrontal cortex, and some executive functions, like abstract thinking, are still evolving during this period (Wachs, Georgieff, Cusick, & McEwen, 2014).

It is possible to determine individual differences in intellectual abilities operationalized as intelligence quotient (IQ). These measures are relatively stable in time, and although intelligence scores may vary during life, they remain strongly correlated (Osler, Avlund, & Mortensen, 2012). Regarding the operationalization of intelligence through the results of intelligence tests, IQ scores can be seen as revealing the individual ability to learn difficult material and the rate at which new material is learned (Torres, 2013). Intelligence tests can also be viewed as measuring learned knowledge, more than general ability. This argument can be based on the finding that one additional year of education added more to the intelligence scores than an additional year of age, suggesting that the measurement of intelligence depends on the acquisition of specific domain knowledge retrieved from long term memory (Tricot & Sweller, 2014). In contrast, it has been posited that IQ scores are only related to academic knowledge as far as knowledge is related to the general intelligence factor, but that the specific intellectual abilities cannot predict achievement further than the g factor (Subotnik et al., 2011). A plausible explanation for the relation between intellectual performance and academic achievement is the posibility of multiple genetically independent brain mechanisms that underly both intellectual performance and learning abilities and create intercorrelations with both of them (Trzaskowski et al., 2013).

Individual differences in intellectual performance, measured as IQ scores predict most of the children´s future achievement, for example, their future success in life when they reach adulthood. It has been found that children and youth with higher IQ scores attain higher levels of education and better jobs, and achieve better economic situation as adults (Firkowska-Mankiewicz, 2011). This is related to the fact that intellectual ability predicts most of the variance of socioeconomic status, like the kind of job an individual can perform well, and therefore the earned income (Torres, 2013).

In sum, even though the origin of individual differences in intelligence or the causal relation between initial individual differences in intelligence and future achievement are still controversial issues, individual differences in IQ scores can validly predict several concurrent academic achievement issues (Subotnik et al., 2011) that are related to future academic and profesional achievements in life. Additionally, intellectual performance can also mediate the relation between adversity and psychological health, and protect the individual against the development of psychopatology resulting from adversity (Flouri, Hickey, Mavroveli, & Hurry, 2011), thus safeguarding the children´s future mental health.

## Predictors of Intellectual Performance 

Beside genetic factors, some environmental aspects (e.g., nutritional, familial, socioeconomic, cultural, and educational) play an important role in the determination of children´s intellectual performance (Tapia et al., 2013). Parental socioeconomic status (SES) affects offspring´s cognitive development in different ways. SES is related to intellectual performance so that children who are raised in better socioeconomic conditions obtain higher IQ scores (midterm effect), and better initial familial economic conditions are related to more successful outcomes later in life (long term effect), despite the children´s initial IQ level (Firkowska-Mankiewicz, 2011).

Family SES may also affect children´s cognitive abilities through nutritional factors, since children from underpriveleged families are exposed to higher risk of malnutrition because of their household precariousness. Adequate nutrition during early childhood is particularly critical to promote healthy brain development, and the required competencies to initiate their school education (Wachs et al., 2014). Early exposition to detrimental nutritional conditions negatively affect the children’s semantic memory, learning, concentration capacity, excutive control, and linguistic performance (Fortunato Araújo, Giatti, Chor, Azeredo, & Barreto, 2014); putting at risk their intellectual, motor, and social development, necessary for academic achievement (Prado & Dewey, 2014).

The effect of the family SES on children’s intellectual performance may be affected by their parent´s intellectual performance, since the positive effect of a better family SES on children´s abilities decreases as maternal intellectual performance increases (Torres, 2013). That is, higher household SES benefits more the children of lower IQ parents, than the children with higher IQ parents, suggesting that being raised by high IQ parents is related to children´s higher intellectual performance, in spite of the household SES. In contrast, the combination of lower household SES and lower IQ parents results in a double disadvantage for the children´s cognitive development (Torres, 2013).

Besides the effect of the household precariousness, children are also exposed to the effect of parental educational attainment, which is usualy low in households with a low SES. Maternal educational attainment is possitively related to their children´s healthier development and better learning environment, with more intellectual stimulation and better academic guidance; all of wich affect the offspring´s cognitive development and their educational attainment (Fortunato Araújo et al., 2014). In this regard, lower parental educational attainment is associated with decrements on their children´s verbal IQ, that affect their cognitive and social functioning (Wachs et al., 2014). So that children from mothers with lower education perform worse on all their cognitive functions and this effect remains during their development into adulthood, in spite of their later educational attainment (Fortunato Araújo et al., 2014).

Some plausible mechanisms of the relationship between household SES and children´s intellectual performance are the insufficient cognitive stimulation the children receive, due to the scarcity of economic resources; as well as the strong association between low parental educational attainment and crystallized inteligence measurements, like verbal IQ (Jensen, Dumontheil, & Barker, 2014). In this sense, higher household SES children are offered more oportunities to benefit themselves from learning experiences more akin to their intellectual interests (Tucker-Drop & Harden, 2012), while parental poverty exposes the children to a stimulation precariousness, that is later associated with the offspring´s lower cognitive funtion (Osler et al., 2012). That is, besides the parental intellectual abilities, the household SES affects the children´s intellectual performance through early nutritional conditions, cognitive stimulation, and parental educational attainment (Fortunato Araújo et al., 2014; Tapia et al., 2013; Torres, 2013).

Additionally, the familial environment in which children grow up also affects their intellectual performance. It has been found that non-authoritarian parenting practices promote the offspring´s intellectual performance (Firkowska-Mankiewicz, 2011). Also parenting practices that emphazise children´s independence, critical thinking, and creativity stimulate the children´s talent development and their motivation to excell (Subotnik et al., 2003). In contrast, a familial environment with high levels of interpersonal stress hinders the development of children´s attention capacity, adversely affecting their intellectual performance and social funtioning (Jensen et al., 2014).

Poverty is associated with negative outcomes on children´s cognitive development and educational achievement (Chaudry & Wimer, 2016). Household poverty negatively affects children´s educational attainment (Parker, Jerrim, Schoon, & Marsh, 2016); so that higher SES is related to higher college enrolment in adolescents (Maxwell, McNeely, & Carboni, 2016), while college enrolment and graduation rates are notoriously lower in communities with higher levels of poverty (Donlan et al., 2016). Some possible mechanisms through which poverty adversely affects educational outcomes are an inadequate development context, deficient cognitive stimulation, and familial stress, as well as the household precariousness (Chaudry & Wimer, 2016). Furthermore, familial adversity, like economic hardships and neighborhood insecurity, is also related to negative academic outcomes, like school failure, through its effect on children´s mental health (Porche, Costello, & Rosen-Reynoso, 2016). In sum, growing up in poverty exposes the children to the risk of nutrimental, economic, and stimulation deficits, which hinder their intellectual development and academic attainment. This underscores the importance of predicting intellectual performance and educational achievement, with the aim of designing possible interventions to promote both in underprivileged youth.

The present study proposes the identification of social and psychological predictors of intellectual performance and educational attainment in a sample of adolescents living in poverty. Age, number of siblings, neighborhood insecurity, and adversity (e.g., unemployment of a family member, conflict between the parents, illness of a family member) were included as social variables, while individual and familial characteristics (e.g., resilience, intern locus of control, parenting practices, and peer relationships) were included as psychological variables. Intellectual performance and educational attainment are the outcomes of interest. The present study hypotheses are: 1) Higher intellectual performance is predicted by better social environment (fewer siblings, less neighborhood insecurity, and less adversity); and by 2) better familial environment (higher maternal intellectual performance and educational attainment, and more adequate parenting practices). Additionally, it was hypothesized that: 3) Higher educational attainment is predicted by higher intellectual performance; and that 4) psychological resources (resilience, intern locus of control, direct coping style, peer relationships, and social support) moderate the relation between adversity (life events) and intellectual performance.

## Method

### Participants

#### Offspring

Data from 1,093 adolescents were analyzed, 61% of them living in urban localities, 55.8% were of male gender, with average age 14.92 (*SD* = 1.29) years, average educational attainment 8.33 (*SD* = 1.71) completed years of education, and average number of siblings 2.67 (*SD* = 1.73).

#### Mothers

Participants were the main beneficiaries of the governmental program against poverty called *Oportunidades*. This is a conditioned cash transference program for households under the line of poverty. The cash transfers are conditioned to children´s health and educational outcomes and the mothers are usually registered as the main beneficiary of the program. Mothers were of average age 40.57 (*SD* = 8.9) years, and average educational attainment 4.66 (*SD* = 3.5) completed years of education.

#### Sample

Access to the national registry of beneficiary households of *Oportunidades* was obtained to extract a random sample of households. Non-Spanish speaking localities, localities with less than 45 households, and households without economic information were excluded from the sampling process. Two-stage sampling was performed for the rural and urban domains separately. Localities were selected through probability proportional to size (PPS) within each domain in the first stage, and households were selected through simple random sampling within each previously selected locality in the second stage. A more detailed description of the sampling design and procedure is presented in Palomar Lever (2015a). A two-wave survey was implemented. The first wave survey resulted in 2,112 households with valid data out of 2,139 surveyed. A further selection of the households with adolescent offspring resulted in a second wave survey with valid data from 1,093 adolescents out of 1,275 initially surveyed.

### Measures

#### Offspring Reported Variables

Validation data on Mexican adolescents of the measures reported by offspring are described more extensively in Palomar Lever (2015b). The descriptive data correspond to the present sample.

#### Intellectual Performance

Adolescent intellectual performance was measured with verbal and nonverbal intelligence tests. The Barranquilla Rapid Survey Intelligence Test BARSIT (del Olmo, 1985) is a verbal test that measures general intelligence and learning aptitude through the intellectual abilities represented in five subtests: General knowledge, Vocabulary, Verbal reasoning, Logical reasoning, and Numerical reasoning (60 items, score = 0–1, Cronbach´s α = .94, range = 0–60, *M* = 31.53, *SD* = 10.29). The Raven´s Progressive Matrices Test (Raven, Raven, & Court, 1993) is a nonverbal test of intelligence that measures general capacity of analogic reasoning, perception and abstraction. The Advanced Scale Series I was used (12 items, score = 0–1, Cronbach´s α = .74, range = 0–12, *M* = 6.57, *SD* = 2.68).

#### Resilience

It refers to the capacity to face and overcome adverse conditions, measured by the factorial scale Resilience (Palomar Lever & Gómez Valdez, 2010), that assesses the perception of personal strength in the face of adversity (14 items, score = 1–4, Cronbach´s α = .86, range = 1–4, *M* = 3.21, *SD* = 0.50).

#### Internal Locus of Control

It refers to the tendency to attribute events to one´s behavior, measured with the factorial scale Locus of Control (Reyes, 1995), which assesses the extent to which one attributes outcomes to one’s own efforts (9 items, score = 1–4, Cronbach´s α = .85, range = 1–4, *M* = 3.39, *SD* = 0.54).

#### Direct Coping Style

Items come from the revised Ways of Coping scale (Folkman & Lazarus, 1985), measuring the tendency to ponder and plan how to solve what causes stress due to unmet environmental demands (6 items, score = 1–4, Cronbach´s α = .72, range = 1–4, *M* = 3.22, *SD* = 0.55).

#### Adversity

The occurrence of adversity was measured through a factorial scale derived from items appropriate to adolescents from the Social Readjustment Rating Scale (Holmes & Rae, 1967). The scale assesses the experience of several adverse life events (13 items, score = 0–1, Cronbach´s α = .71, range = 0–1, *M* = 0.15, *SD* = 0.16).

#### Neighborhood Insecurity

This was measured with items from another instrument (Molnar, Gortmaker, Bull, & Buka, 2004), which assess the perception of one´s neighborhood as insecure and dangerous (12 items, score = 1–4, Cronbach´s α = .91, range =1–4, *M* = 2.04, *SD* = 0.72).

#### Parenting Practices

The tendency to apply parenting practices was assessed with several subscales from the Parenting Practices scales (Andrade & Betancourt, 2010) like Paternal practices of psychological control (8 items, score = 1–4, Cronbach´s α = .84, range =1–4, *M* = 2.72, *SD* = 0.92), Paternal practices of imposition (8 items, score = 1–4, Cronbach´s α = .81, range = 1–4, *M* = 1.44, *SD* = 0.59), and Maternal practices of imposition (8 items, score = 1–4, Cronbach´s α = .76, range = 1–4, *M* = 3.09, *SD* = 0.74). These subscales measure the parental tendencies to use coercive methods of discipline and to impose their criteria over those of their children.

#### Permissive Parental Style

This factorial scale was derived from other instruments (Arnold, O'Leary, Wolff, & Acker, 1993; Robinson, Mandelco, Frost, & Hart, 1995), and assesses the parental tendency to be inconsistent in applying disciplinary actions and to allow and tolerate their offspring´s disruptive behavior (6 items, score = 1–4, Cronbach´s α = .57, range = 1–4, *M* = 1.94, *SD* = 0.56).

#### Social Support

This factorial scale (Zimet, Dahlem, Zimet, & Farley, 1998) assesses the perception of the availability of people in their environment who give them support, encouragement, and understanding (12 items, score = 1–4, Cronbach´s α = .93, range = 1–4, *M* = 3.28, *SD* = 0.64).

#### Positive Peer Relationships

This factorial scale (DuBois, Felner, Brand, Phillips, & Lease, 1996) assesses the self-perception as gregarious and popular among their peers and friends (7 items, score = 1–4, Cronbach´s α = .83, range = 1–4, *M* = 3.12, *SD* = 0.60). Additionally, the adolescents´ age, educational attainment, and the number of siblings was also recorded.

#### Mother Reported Variables

The validation data in Mexican adults of the measurements assessed in the mothers of the adolescents are described in Palomar Lever (2015a). The descriptive data refer to the present sample.

#### Intellectual Performance

The same verbal and nonverbal intelligence tests applied to adolescents were used to assess mother´s intellectual performance: the Barrranquilla Rapid Survey Intelligence Test BARSIT (del Olmo, 1985), (60 items, score = 0–1, Cronbach´s α = .95, range = 0–60, *M* = 27.90, *SD* = 11.26), and the Raven´s Progressive Matrices Test (Raven et al., 1993), (12 items, score = 0–1, Cronbach´s α = .78, range = 0–12, *M* = 4.50, *SD* = 3.0). Additionally, maternal educational attainment was also recorded.

### Procedure

Beneficiary mothers and their adolescent offspring were assessed separately using two different surveys. Mothers were assessed in the first survey and their offspring in the second one. In both surveys participants were interviewed face to face individually at their homes by professional interviewers using questionnaires. Interviews lasted approximately an hour. Informed consent was obtained from mothers and informed consent and permission was obtained from adolescent´s mothers to authorize their adolescent offspring´s participation. Participants were informed that their participation was voluntary and independent from their program involvement and were assured of their data confidentiality.

## Results

Covariance analysis of the dependent variables of interest (intellectual performance and educational attainment) by gender and type of locality, while controlling for age, indicated that there were no significant differences in verbal and nonverbal intelligence scores by gender, nor were there significant differences in nonverbal intelligence scores or educational attainment by type of locality (*p* > .066 ). However, significant differences were found in verbal intelligence scores by type of locality: *M*_rural_ = 30.66, *SD* = 10.29, *n* = 426, *M*_urban_ = 32.09, *SD* = 10.23, *n* = 667, *F*(1,1090) = 4.62, *p* = .032, $\eta_{p}^{2}$ = .004, and in educational attainment by gender: *M*_men_ = 8.19, *SD* =1.79, *n* = 610, *M*_women_ = 8.52, *SD* = 1.58, *n* = 483, *F*(1,1090) = 14.03, *p* < .001, $\eta_{p}^{2}$ = .013; favoring urban and feminine adolescents, respectively.

The data reported by the adolescents and their mothers were analyzed with structural equations modeling (SEM) using the maximum likelihood method. SEM is a multivariate statistical analysis technique that can combine factor analysis and path analysis to define latent variables using observed variables, and to test structural models that impute relationships between latent variables.

One model was fitted to the entire sample, since the aforementioned covariance analyses did not show generalized group differences. The tested model consisted of latent variables like *Offspring Intellectual Performance* (verbal and nonverbal intelligence test scores), *Inadequate Parenting* (permissive parental style, paternal practices of psychological control and imposition and maternal practices of imposition), *Maternal Intellectual Performance and Education* (verbal and nonverbal intelligence test scores and educational attainment), and *Offspring Psychological Resources* (resilience, internal locus of control, direct coping strategies, positive peer relations, and social support). Adolescent´s age, number of siblings, adversity (life events), and their perception of neighborhood insecurity were also included as predictors. The model tested the prediction of adolescent´s intellectual performance and educational attainment.

The tested model showed that higher offspring intellectual performance was predicted by older age, higher maternal intellectual performance and education, more adversity, fewer siblings, lower levels of inadequate parenting, and lower levels of neighborhood insecurity. Further, higher offspring educational attainment was predicted by older age, higher intellectual performance, higher levels of psychological resources and lower levels of inadequate parenting. Additionally, higher levels of psychological resources were predicted by higher intellectual performance. Finally, lower levels of maternal intellectual performance and education covaried with higher levels of inadequate parenting, and older age and higher neighborhood insecurity covaried with more adversity. No significant covariance was found between adversity and psychological resources (*p* = .077).

**Figure 1:**
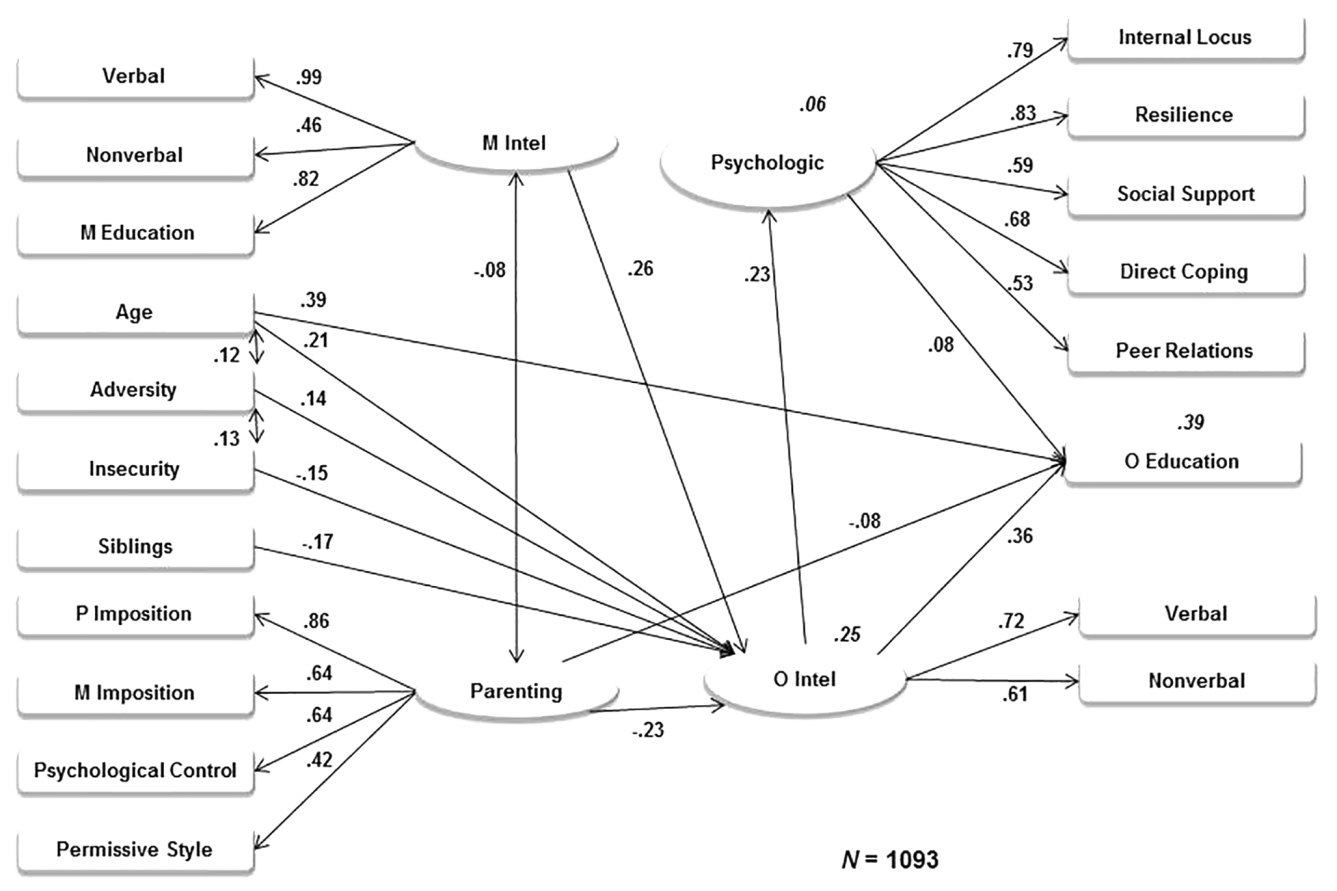
Modeling of familial, social, and psychological predictors of adolescent´s intellectual performance and educational attainment. *Note*. All coefficients were significant. Errors are not depicted and *R*^2^ coefficients are depicted in italics. Verbal = BARSIT; Nonverbal = Raven; M Education = Maternal educational attainment; M Intel = Maternal intellectual performance and education; Adversity = Adverse life events; Insecurity = Neighborhood insecurity; Siblings = Number of siblings; P Imposition = Paternal parental practices of imposition; M Imposition = Maternal parental practices of imposition; Psychological Control = Paternal parental practices of psychological control; Permissive Style = Permissive parental style; Parenting = Inadequate parental practices; Psychologic = Psychological resources; O Intel = Offspring intellectual performance; Internal Locus = Internal locus of control; Direct Coping = Direct coping strategies; Peer Relations = Positive peer relations; O Education = Offspring educational attainment.

The model attained an acceptable fit (Hu & Bentler, 1999), taking into account its complexity and the sample size: χ^2^(143) = 559.35, *p* < .001, CFI = 0.922, TLI = 0.907, RMSEA = 0.052, 90% CI [0.047, 0.056], *p*_close_ = .264. The variables included in the model explained approximately 25% of the variance of the offspring intellectual performance, 39% of the variance of their educational attainment, and 6% of their psychological resources, corresponding to *R*^2^ coefficients of 0.245, 0.388, and 0.055, respectively. The Figure 1 shows the model with the standardized regression and correlation coefficients. All coefficients were significant at the *p* < .05 level.

## Discussion

The objective of the present analysis was to identify predictors of the intellectual performance and educational attainment of youth living in poverty. To this effect, data from adolescents and their mothers, beneficiaries of a governmental program against poverty, were analyzed. Early analyses showed few differences by gender and type of locality controlling by age in favor of women´s educational attainment, and of urban adolescents´ verbal intelligence scores. Additionally, the SEM analysis indicated that the proposed model predicted both the adolescent´s intellectual performance and educational attainment.

The gender difference in educational attainment is consistent with previous results indicating positive effects of the *Oportunidades* program on female educational attainment (Darney et al., 2013). And the difference in verbal intellectual abilities by type of locality is in line with previous data stressing the disadvantage concerning academic resources that youth living in rural environments are confronted with (Culliney, 2014).

Regarding the proposed hypotheses, results partially supported the first hypothesis, since fewer siblings and less neighborhood insecurity, but not less adversity (e.g., better social environment), predicted higher intellectual performance. Contrary to what was expected, more adversity (life events) predicted higher intellectual performance. Data supported the second hypothesis, since higher maternal intellectual performance and education and a lower level of inadequate parenting practices (e.g., better familial environment) predicted higher offspring intellectual performance. Also the third hypothesis received empirical support, since higher intellectual performance predicted higher educational attainment by the adolescents. However, the fourth hypothesis was not supported by the data, since psychological resources do not moderate the effect of adversity on intellectual performance. On the contrary, there was no relation between psychological resources and adversity, while adversity positively predicted intellectual performance, and intellectual performance positively predicted psychological resources. A possible explanation could be that adolescents with higher intellectual performance are more aware of stressful events and have more psychological resources to counteract the negative impact of adversity.

One of the stronger predictors of adolescent´s intellectual performance and educational attainment was their age. This relation is not surprising because the present sample comprises young people in academic formation, who have not yet reached their full intelligence potential nor have they completed their educational instruction. As has been previously found, an additional year of age is associated with increases in youths’ intellectual performance and educational attainment (Tricot & Sweller, 2014).

Present results are congruent with others that signal the close and positive relationship between intellectual performance and various aspects of academic achievement and educational achievement (Subotnik et al., 2011; Tricot & Sweller, 2014), as well as with those that underscore the positive influence of maternal educational attainment (Fortunato Araújo et al., 2014; Wachs et al., 2014), and maternal intellectual performance (Torres, 2013) on their offspring´s intellectual performance. Results are also consistent with other analyses that highlight the importance of parental intellectual wealth on the development of their offspring´s intellectual abilities (Firkowska-Mankiewicz, 2011; Torres, 2013), and their educational attainment (Fortunato Araújo et al., 2014), emphazising the importance of the richness of the household´s knowledge base in conditions of high levels of poverty.

Inadequate parenting practices like coercion, imposition, permissiveness and inconsistent disciplinary practices, showed an inverse relationship with offspring´s intellectual performance. This underlines the importance of the familial evironment in which the adolescents are growing up, and concours with other results documenting the adverse effect of coercive practices on the cognitive development of children (Jensen et al., 2014). Present data indicated that lower maternal intellectual performance and education covaries with higher levels of inadequate parental practices such that low maternal education has a double negative effect on offspring´s intellectual performance: the effect due to the possible insufficiency of maternal crystallized intelligence, and the effect through the promotion of inadequate parental practices.

Two social variables, number of siblings at home and neighborhood security, were directly related to offspring intellectual performance. A greater number of siblings predicted lower intellectual performance, a result that may be associated with the availability of parental economic, cultural, and time resources which decrease, as the number of children among whom parents must divide household resources increasses. Furthermore, a social stressor like neighborhood insecurity negatively predicted adolescent´s intellectual performance, consistent with other results documenting the negative effect of insecurity on youth´s cognitive and academic development (Johnson, 2008).

Life events were included in the model as an adversity measure with the purpose of taking into account the possibility of adversity affecting intellectual performance (Fortunato Araújo et al., 2014; Osler et al., 2012). Data showed that youth who were more exposed to adversity also achieved a higher intellectual performance. This result is unexpected and at odds with the asumption that adversity negatively affects intellectual performance through the stress it causes (Jensen et al., 2014) and through its negative effects on psychological health (Porche et al., 2016). On the contrary, experiencing more adversity seems to incentivize intellectual performance. While this result seems counterintuitive, it is consistent with the view that difficult home environments may lead some children to seek refuge in intellectual and creative activities (Subotnik et al., 2003); thus improving their crystallized intelligence.

Similarly, psychological resources were proposed as moderators of the possible negative effects of adversity on intellectual performance. Results indicated that higher intellectual performance predicted higher levels of psychological resources, consistent with other results previously pointing to this positive relation (Shoshani & Slone, 2013). However, the experience of adversity had no relation with psychological resources, which is inconsistent with their proposed moderating role. On the contrary, data revealed that adversity increases intellectual performance, and this in turn increases psychological resources. That is, intellectual performance mediates between adversity and psychological resources, and the psychological resources do not mediate the relationship between adversity and intellectual performance, as was initially proposed.

It has been previously found that intellectual performance may moderate the negative impact of adversity (Flouri et al., 2011); but present data rather suggest a catalytic role for adversity, as if it would encourage adolescents to achieve higher intellectual performance. A closer look into the correlations between age, neighborhood insecurity, and adversity reveals that they were in the expected direction. That is, older age and higher levels of neighborhood insecurity covaried with more adversity, so an error measurement seems less plausible as an explanation for the present results. More research would be necessary to explain the relationship between adversity, psychological resources, and intellectual performance.

In sum, results are congruent with the proposition that the intellectual performance of adolescents living in poverty is positively predicted by familial environments with a richer parental knowledge base, less coercive parenting, and fewer siblings, and by less insecure neighborhoods. Adolescents’ educational attainment was positively predicted by higher intellectual performance, greater psychological resources, and less coercive parenting. Additionally, the adolescents´ age had an important role in the predictition of their intellectual performance and educational attainment.

### Limitation and Future Direction

The present study provides information about a less studied population sector based on data from a national probabilistic sample of households beneficiaries of a government program against poverty. However, some weaknesses of the study, like the crosssectional design of the study and the use of self-reports as measurements of some variables, should also be mentioned.

Our data on a national sample of households enable the present results to be extended to other benefiaries of the program nationwide, and to design strategies tailored to this population sector, with the purpose of preventing the transgenerational transmission of poverty. Present results allow the suggestion of possible intervention strategies aimed at enhancing the intellectual performance of adolescents living in poverty and supporting their educational attainment.

Some of these possibilities encompass the implementation of informative actions directed to parents about the deleterious effects of inadequate parenting practices and how to avoid them, as well as government actions to reinforce public security. An additional strategy is to promote parental educational attainment. There is already a government institution dedicated to adult education, so that the promotion of educational attainment of mothers beneficiaries of the *Oportunidades* program would be advantageous for them and their children, taking into account the possible positive effects of parental education on the intellectual performance of their offspring.
